# Singular cases of Alzheimer’s disease disclose new and old genetic “acquaintances”

**DOI:** 10.1007/s10072-020-04774-y

**Published:** 2020-10-02

**Authors:** Cinzia Coppola, Dario Saracino, Mariano Oliva, Lorenzo Cipriano, Gianfranco Puoti, Sabina Pappatà, Giuseppe Di Fede, Marcella Catania, Martina Ricci, Sara Cimini, Giorgio Giaccone, Simona Bonavita, Giacomina Rossi

**Affiliations:** 1 Department of Advanced Medical and Surgical Sciences, University of Campania “L. Vanvitelli”, Naples, Italy; 2 Second Division of Neurology, University of Campania “Luigi Vanvitelli”, Isola 8 – Edificio 10 Policlinico “Federico II” via Pansini 5, 80131 Naples, Italy; 3 Institute of Biostructure and Bioimaging, National Council of Research, Naples, Italy; 4 Department of Advanced Biomedical Sciences, Federico II University, Naples, Italy; 5 Division of Neurology V – Neuropathology, Fondazione IRCCS Istituto Neurologico Carlo Besta, Milan, Italy

**Keywords:** Alzheimer’s disease, Mutation, Dementia, Amyloid, Genetics, Biomarkers

## Abstract

**Background:**

Alzheimer’s disease (AD) is the most common age-related dementia. Besides its typical presentation with amnestic syndrome at onset, atypical AD cases are being increasingly recognized, often in presenile age.

**Objectives:**

To provide an extensive clinical and genetic characterization of six AD patients carrying one or more singular features, including age of onset, atypical phenotype and disease progression rate. By reviewing the pertinent literature and accessing publicly available databases, we aimed to assess the frequency and the significance of the identified genetic variants.

**Methods:**

Biomarkers of amyloid-β deposition and neurodegeneration were used to establish the in vivo diagnosis of probable AD, in addition to neurological and neuropsychological evaluation, extensive laboratory assays and neuroradiological data. Considering the presenile onset of the majority of the cases, we hypothesized genetically determined AD and performed extensive genetic analyses by both Sanger sequencing and next generation sequencing (NGS).

**Results:**

We disclosed two known missense variants, one in *PSEN1* and the other in *PSEN2*, and a novel silent variant in *PSEN2*. Most notably, we identified several additional variants in other dementia-related genes by NGS. Some of them have never been reported in any control or disease databases, representing variants unique to our cases.

**Conclusions:**

This work underlines the difficulties in reaching a confident in vivo diagnosis in cases of atypical dementia. Moreover, a wider genetic analysis by NGS approach may prove to be useful in specific cases, especially when the study of the so-far known AD causative genes produces negative or conflicting results.

## Introduction

Alzheimer’s disease (AD) is the most common age-related degenerative dementia. From a clinical perspective, AD typically displays an amnestic syndrome of the hippocampal type that can be associated with various cognitive or behavioural deficits during disease evolution [1]. Atypical forms of AD present with relative preservation of memory at onset and generally occur at an earlier age. They include posterior, logopenic and frontal variant of AD [1]. According to the revised international criteria, at least one biomarker of in vivo Alzheimer’s pathology must be positive: a cerebrospinal fluid (CSF) profile consisting of decreased amyloid-β 1–42 (Aβ_42_) together with increased total tau (T-tau) or 181-phopshorylated tau (P-tau) concentrations, or an increased retention on amyloid tracer PET (AMY-PET) [1]. In addition to the use of single CSF markers, the combination of multiple CSF markers in the form of ratios further increases the diagnostic accuracy [2]. AD is usually sporadic, with age of onset most often being > 65 years, thus qualifying for late onset AD (LOAD). In no more than 5% of all patients, a positive familial history for dementia or a clear-cut autosomal dominant pattern of inheritance can be found. These familial AD cases (FAD) arise before age 65 more frequently than sporadic cases, hence the definition of early onset AD (EOAD) [3]. Approximately 50% of FAD patients carry a mutation in presenilin 1 (*PSEN1*), presenilin 2 (*PSEN2*) or amyloid-β protein precursor (*APP*) genes, with more than 350 variants collectively identified so far [4, 5]. However, some of them are not pathogenic or their significance remains uncertain, as they may qualify as genetic risk factors or disease-modifying alterations.

Here, we describe a case series of cognitive disorders with in vivo biomarker positivity for Aβ deposition, showing various clinical atypical aspects together with peculiar genetic features. We disclosed two known missense variants, one in *PSEN1* and the other in *PSEN2*, and a novel silent variant in *PSEN2*. Moreover, additional variants in dementia-related genes have been identified by next generation sequencing (NGS). Our results are intriguing as they raise the question of the role of genetic risk burden in AD.

## Patients and methods

### Subjects

We describe 6 unrelated cases affected by cognitive disorders who underwent a complete diagnostic protocol including neurological and neuropsychological evaluation, extensive laboratory assays, EEG, structural (CT or MR) and functional (^18^FDG-PET) neuroimaging. The research for AD pathophysiological biomarkers, either CSF Aβ_42_, T-tau and P-tau assay or amyloid tracer PET, was performed in all patients.

#### Case 1

**Fig. 1:**
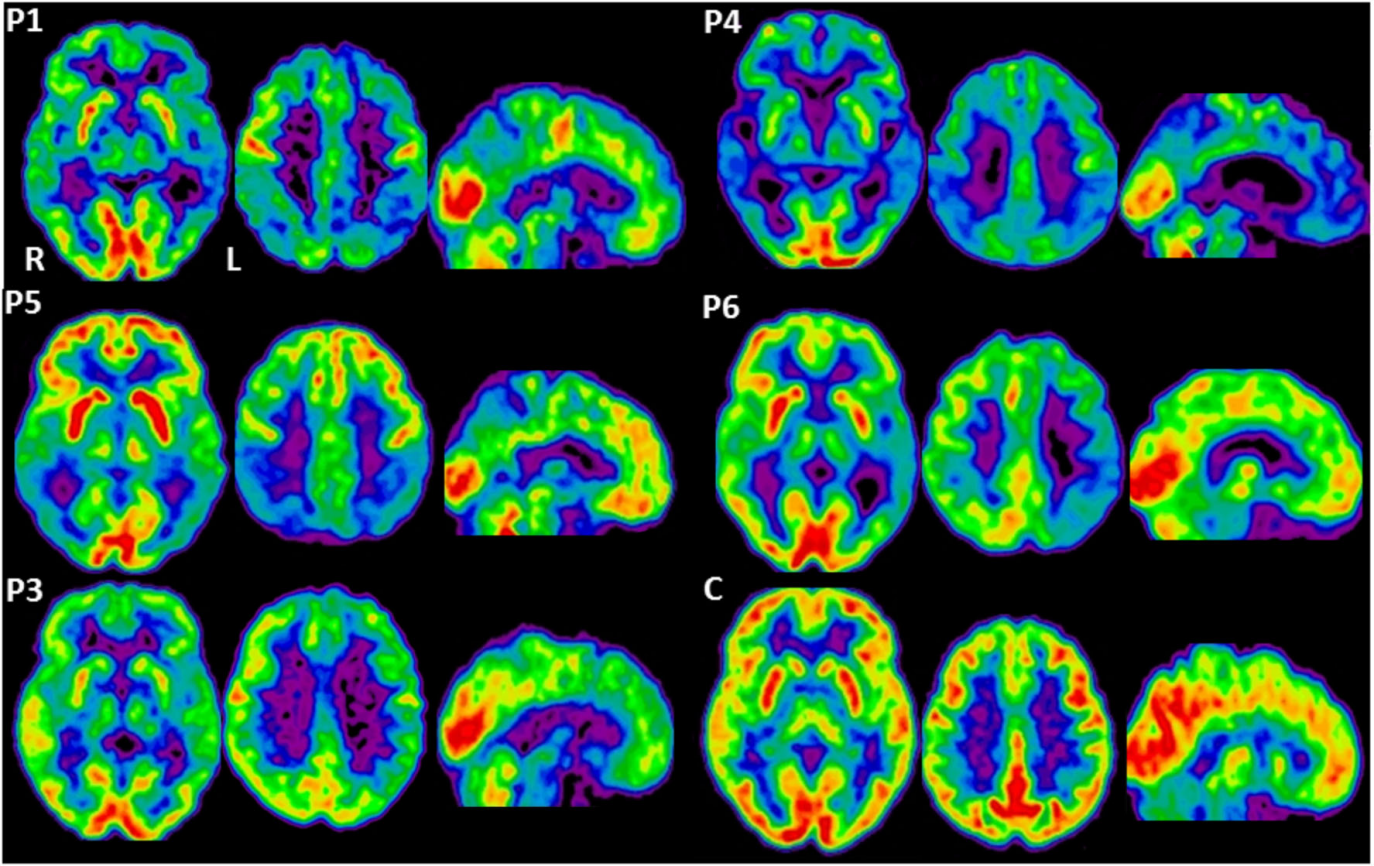
PET transaxial images at two axial and one sagittal section show ^18^FDG uptake in the patients (P1, P4, P5, P6, P3) and in a control subject (C). Compared with control, P1, P4, and P5 showed a relative hypometabolism involving mainly the posterior cortical regions including the PCC with some spread to the frontal cortex in P1, P4 and to the striatum (R < L) in P4. P6 and P3 show less marked asymmetric hypometabolism (L < R) involving also the striatum (L < R)

This patient insidiously presented at age 55 with short-term memory impairment and apathy. Familial history and neurological examination were negative, except for Epstein sign; MMSE was 22/30. An extensive neuropsychological evaluation showed deficits of long- and short-term memory, language, abstract reasoning, executive functions and a marked anosognosia. Brain MRI disclosed diffuse cortical atrophy, while ^18^FDG-PET (Fig. 1) revealed bilateral hypometabolism in the frontal dorso-lateral, superior parietal, temporo-parietal cortices, with prevalent involvement of the left hemisphere, and posterior cingulate cortex (PCC). AMY-PET showed increased uptake mainly in the frontal and lateral temporal regions.

#### Case 2

In this patient, the onset of cognitive impairment was approximately at 75 years and characterized by short-term memory deficits, anomia, subtle behavioural changes (mild disinhibition) and in the following months psychomotor slowing. Familial history was negative. Neurological examination disclosed an asymmetric parkinsonian syndrome (R > L). MMSE was 22/30. Neuropsychological testing revealed deficits in verbal memory, attention, abstract reasoning and semantics. Brain MRI showed moderate atrophy in frontal, lateral temporal and temporo-mesial cortex prevailing on the left side. AMY-PET evidenced a massive and diffuse burden of amyloid-β. His extrapyramidal syndrome showed satisfying response to L-Dopa administration.

#### Case 3

This woman presented with apathy and short-term memory deficit at the age of about 62. Family history evidenced memory disturbances in her mother and grandmother. MMSE was 27/30. Neuropsychological assessment detected long-term verbal memory and attentional deficits. Brain CT revealed diffuse supratentorial white matter hypodensity, while ^18^FDG-PET (Fig. 1) showed mild hypometabolism mainly affecting the left hemisphere and involving the mesial and lateral temporal cortex, the dorsolateral/medial frontal cortex and to a lesser extent the PCC. AMY-PET evidenced a diffuse amyloid deposition.

#### Case 4

This patient, without family history of dementia, around the age of 59 developed apathy with a language disorder characterized by word-finding problems and slow, hesitating speech, followed by psychomotor agitation, delusional ideation, clumsiness of his upper left limb and generalized motor slowness. His language got significantly worse, with agrammatism and telegraphic sentences, but with only mild impairment in comprehension. Neurological examination at age 61 disclosed mixed pyramidal and extrapyramidal syndrome, prevailing on the left side, left cortical sensory loss and frontal release signs. His MMSE score was 7/30, being non-fluent aphasia with features of apraxia of speech and dressing apraxia among the most significant cognitive deficits. Brain MRI revealed discrete atrophy mainly in temporo-insular cortices bilaterally, whereas ^18^FDG-PET (Fig. 1) disclosed severe and diffuse cortical hypometabolism more marked in temporo-parietal cortices, precuneus and PCC bilaterally, with prevalent involvement of the right side and slight striatal metabolic asymmetry (R < L). AMY-PET detected diffuse burden of amyloid-β.

#### Case 5

This subject developed at age 45 a complex behavioural syndrome characterized by apathy, social withdrawal, and eating and sleep disorders. Familial history was negative. Over the next 5 years, there was a clinical worsening with word-finding difficulties, dyscalculia, memory deficits and motor clumsiness in both hands. At age 50, his MMSE score was 16/30 and he presented ideo-motor apraxia, anomia, verbal memory deficits and dysexecutive syndrome. EEG showed diffuse slowing of cerebral electric activity, while brain MRI detected atrophy in parietal regions with slight right prevalence. Subsequently, he also manifested limb myoclonus and psychomotor agitation with complex visual hallucinations. Seven years after the symptom onset, the patient came to our observation and underwent a more extensive diagnostic protocol. Neurological examination showed left pyramidal and bilateral asymmetric (L > R) extrapyramidal syndrome, action-induced limb myoclonus and Epstein sign. MMSE score was 8/30. Brain MRI evidenced marked and diffuse atrophy, with posterior predominance. ^18^FDG-PET (Fig. 1) demonstrated bilateral hypometabolism in the parietal, occipital and temporal lobes and in the PCC with relative sparing of frontal lobes and subcortical structures. The occipital hypometabolism mostly involved the associative visual regions with relative sparing of the primary visual cortex. CSF biomarkers assay revealed reduced Aβ_42_ (456 pg/mL; normal values – n.v. – > 500 pg/mL) [6] and a massive increase of both T-tau (3435 pg/mL; n.v. < 300 pg/mL) [6] and P-tau (470 pg/mL, n.v. < 61 pg/mL) [7]. T-tau/Aβ_42_ ratio was 7.533 (n.v. ≤ 0.52), whereas P-tau/Aβ_42_ was 1.031 (n.v. ≤ 0.08) [2].

#### Case 6

This case, without family history for cognitive disorders, insidiously presented at age 52 with a language disorder characterized by anomia and apraxia of speech which progressively worsened until mutism. Neurological examination showed a “worried” facial expression, asymmetric (R > L) mixed pyramidal and extrapyramidal syndrome, focal and segmental myoclonus, exaggerated startle reaction and frontal release signs. A neuropsychological examination showed severe non-fluent aphasia with almost complete mutism and slightly impaired comprehension, severe bucco-lingual and ideo-motor apraxia. EEG showed marked slowing in cerebral electric activity. Brain MRI revealed asymmetrical cortico-subcortical atrophy prevailing in left fronto-temporal areas. ^18^FDG-PET (Fig. 1) showed asymmetric cortical hypometabolism characterized by a prevalent involvement of the left temporo-parietal cortex and, to a lesser extent, of the left premotor-motor and sensorimotor regions. In addition, there was also a mild left striatal and thalamic hypometabolism. CSF Aβ_42_ was decreased (235 pg/ml; n.v. > 500 ng/mL), whereas T-tau and P-tau were normal; T-tau/Aβ_42_ and P-tau/Aβ_42_ ratios were 1.247 and 0.145 respectively. The research of 14.3.3 protein in CSF was negative.

### Patient consents

Written informed consent was acquired from all patients for genetic analysis, processing data and permission to publish data in respect of privacy.

### Biochemical analysis

CSF levels of T-tau, P-tau and Aβ_42_ were determined with human specific ELISA kits (Innogenetics). Plasma level of progranulin was measured using an ELISA kit (Human Progranulin ELISA kit, Adipogen Inc., Seoul, Korea).

### Genetic analysis

Sanger Sequencing of *APP*, *PSEN1* and *PSEN2* genes [8, 9] and *APOE* genotyping [10] was performed in all cases. Additionally, a gene panel of 48 dementia-related genes was analysed by NGS techniques. Nextera Rapid Capture system for enrichment (Illumina) coupled with gene-specific probes (Integrated DNA Technologies) was used to sequence the following genes: *APP*, *PSEN1*, *PSEN2*, *PRNP*, *GRN*, *MAPT*, *CHMP2B*, *FUS*, *TARDBP*, *VCP*, *TREM2*, *ABCA7*, *APOE*, *BIN1*, *CALHM1*, *CCL2*, *CCNF*, *CD33*, *CHCHD10*, *CLU*, *CSF1R*, *CST3*, *CTSF*, *DCTN1*, *FLNC*, *hnRNPA1*, *hnRNPA2B1*, *ITM2B*, *LRRK2*, *NCSTN*, *NOS3*, *NOTCH3*, *OPTN*, *PFN1*, *PLD3*, *PRKAR1B*, *SERPINI1*, *SIGMAR1*, *SNCA*, *SNCB*, *SORL1*, *SQSTM1*, *STH*, *TBK1*, *TMEM106B*, *TUBA4A*, *TYROBP*, *UBQLN2*. Sequencing was performed on the Illumina MiSeq instrument using 2X150 bp paired-end read cycles. MiSeq Reporter software (Illumina) was used for alignment (reference human genome UCSC hg19) and variant calling. Variants were annotated using Variant Studio software (Illumina). Low-quality variants were filtered out using the Illumina Qscore threshold of 30; in addition, variants with a minor allele frequency higher than 2% in GnomAD (Genome Aggregation Database, http://gnomad.broadinstitute.org/) were filtered out. Variants of interest were confirmed using standard Sanger sequencing.

Sorting Intolerant From Tolerant (SIFT) and Polymorphism Phenotyping (PolyPhen) softwares were used to predict pathogenicity of missense mutations. Combined annotation-dependent depletion (CADD) score (https://cadd.gs.washington.edu/) was used to predict the pathogenicity of a truncating variant (*SORL1* Ser10STOP). NetGene2 (http://www.cbs.dtu.dk/services/NetGene2/) and BDGP (http://www.fruitfly.org/seq_tools/splice.html) splice site prediction tools were used to predict the effect on the splice site of the *DCTN1* c.3529 + 5G > A variant.

## Results

### Clinical, instrumental and CSF findings

Our series consists of six cases whose clinical features are summarized in Table 1. Disease onset was in the presenile period in all patients (mean age of onset: 54.6 ± 6.6), except for case 2. Only case 3 showed family history for dementia. The onset was typical in two patients (cases 1 and 3) and atypical in the others. Moreover, an extrapyramidal syndrome complicated all these atypical cases. The clinical diagnosis was AD in cases 1, 2, 3 and 4. In case 5, there was an important discrepancy between clinical findings, suggestive of behavioural variant of frontotemporal dementia (bvFTD) with parkinsonism, and MRI and ^18^FDG-PET data, expression of atypical AD. In case 6, the clinical diagnosis was corticobasal syndrome (CBS). Given the peculiarity of disease onset, plasma progranulin dosage was performed in cases 4, 5 and 6, with normal values. In addition, all patients underwent an *APOE* genotyping, which only in cases 2 and 3 showed a ε3/ε4 heterozygosity. The diagnosis of probable AD was supported by at least one positive pathophysiological biomarker in all cases: AMY-PET in cases 1, 2, 3 and 4 and CSF biomarkers in cases 5 and 6. In case 6, although T-tau and P-tau values were not increased, T-tau/Aβ_42_ and P-tau/Aβ_42_ ratios both resulted well above the standardized cut-offs [2], thus strongly suggesting an underlying AD pathology.

**Table 1:** Clinical, instrumental and laboratory data of patients

Case	Age at onset (y)	Cognitive symptoms at onset	Motor syndrome	MRI/CT	^18^FDG-PET	Amyloid tracer PET	CSF biomarkers					Plasma progra-nulin	*APOE*	Diagnosis
							Aβ_42_	T-tau	P-tau	T-tau/ Aβ_42_	P-tau/ Aβ_42_			
1	55	Memory and attention disorders	Absent	Diffuse cortical atrophy	Bilateral temporo-parietal, precuneus, PCC and frontal dorso-lateral cortical hypometabolism (L < R)	Increased cortical uptake in the frontal and lateral temporal regions	n.a	n.a.	n.a.	n.a.	n.a.	n.a.	3/3	EOAD
2	75	Memory deficit and behavioural syndrome	Asymmetric parkinsonism (R > L) L-Dopa responsive	Asymmetric fronto-temporal atrophy with left prevalence	n.a.	Marked and diffuse uptake	n.a	n.a.	n.a.	n.a.	n.a.	n.a.	3/4	Atypical LOAD
3	62	Apathy and memory deficit	Absent	Diffuse supratentorial white matter hypodensity	Mild left mesial and lateral temporal and frontal cortices hypometabolism; very mild left PCC and putamen hypometabolism	Increased cortical uptake in the frontal, lateral temporal and parietal regions	n.a	n.a.	n.a.	n.a.	n.a.	n.a.	3/4	Familial EOAD
4	59	Apathy, apraxia, non-fluent aphasia	Mixed pyramidal and extrapyramidal syndrome (L > R)	Temporo-insular atrophy	Marked diffuse bilateral cortical hypometabolism more evident in the temporo-parietal cortex, precuneus and PCC (R < L); slight striatal hypometabolism (R < L)	Diffuse uptake	n.a.	n.a.	n.a.	n.a.	n.a.	148.9	3/3	Atypical EOAD
5	45	Behavioural syndrome, sleep disorder	Left pyramidal and asymmetric extrapyramidal syndrome (L > R); myoclonus	Bilateral posterior (mainly parietal) cortical atrophy	Marked bilateral temporal, parietal, associative occipital cortex and PCC hypometabolism.	n.a.	456	3435	470	7.533	1.031	135.6	3/3	Atypical EOAD
6	52	Speech disorders	Mixed pyramidal and extrapyramidal syndrome (R > L); myoclonus	Asymmetrical cortico-subcortical atrophy with left fronto-temporal prevalence	Cortical temporal, parietal and frontal premotor and sensorimotor hypometabolism (L < R); mild left striatal, thalamic and PCC hypometabolism	n.a.	235	293	34	1.247	0.145	100.3	3/3	Atypical EOAD presenting as CBS

*EOAD*, early-onset Alzheimer’s disease; *LOAD*, late-onset Alzheimer’s disease; *n.a.*, not available; *PCC*, posterior cingulate cortex; *y*, years; *CBS*, cortico-basal syndrome

### Genetic findings

Diagnostic genes (*APP*, *PSEN1*, *PSEN2*, *PRNP*, *GRN*, *MAPT*, *CHMP2B*, *FUS*, *TARDBP*, *VCP*, *TREM2*) were sequenced at 100% by NGS (read depth ≥ 20X) or, in some cases with incomplete coverage, by standard Sanger technique. Genetic results are described in Table 2 and Table 3. Population frequency, in silico pathogenicity prediction (SIFT and Polyphen) and classification in Human Gene Mutation Database (HGMD) are presented. Briefly, concerning AD-causative genes (Table 2), we identified two known missense variants, Glu318Gly in *PSEN1* (patients 1 and 2) and Arg71Trp in *PSEN2* (patient 3), and a novel silent variant, Ser236Ser in *PSEN2* (patient 4). Moreover, thanks to NGS approach, we disclosed other variants in dementia-related genes, in particular *FUS*, *ABCA7*, *CSF1R*, *DCTN1*, *SERPINI1* and *SORL1* (Table 3).

**Table 2:** DNA variants found in genes causative for AD

Case	Gene	Coordinates	Transcript	DNA variant	Amino acid variant	Sift	PolyPhen	GnomAD Freq %	HGMD classification
1	*PSEN1*	73,673,178	NM_000021.3	gAa/gGa	Glu318Gly	tol	ben	1.485	Disease-associated polymorphism with supporting functional evidence
2	*PSEN1*	73,673,178	NM_000021.3	gAa/gGa	Glu318Gly	tol	ben	1.485	Disease-associated polymorphism with supporting functional evidence
3	*PSEN2*	227,071,475	NM_000447.2	Cgg/Tgg	Arg71Trp	del	ben	0.3836	Disease causing mutation?
4	*PSEN2*	227,076,671	NM_000447.2	agT/agC	Ser236Ser	-	-	1.331	No

*Sift*, Sorting Intolerant From Tolerant software; *PolyPhen*, Polymorphism Phenotyping software; *GnomAD*, Genome Aggregation Database; *HGMD*, Human Gene Mutation Database; *ben*, benign; *del*, deleterious; *tol*, tolerated

**Table 3:** DNA variants found in other dementia-related genes

Case	Gene	Coordinates	Transcript	DNA variant	Amino acid variant	Sift	PolyPhen	GnomAD Freq %	HGMD classification
1	*ABCA7*	1047345	NM_019112.3	Gac/Tac	Asp679Tyr	del	prob dam	0	No
	*SORL1*	121425954	NM_003105.5	aCa/aTa	Thr833Ile	del	poss dam	0	No
2	*FUS*	31196452	NM_004960.3	tAt/tGt	Tyr239Cys	tol	prob dam	0.001698	No
5	*DCTN1*	74598723	NM_004082.4	Atc/Gtc	Ile196Val	tol	ben	0.4519	Functional polymorphism
	*SORL1*	121323069	NM_003105.5	tCg/tAg	Ser10STOP*	-	-	0	No
6	*CSF1R*	149456911	NM_005211.3	Gcc/Acc	Ala273Thr	tol	prob dam	0	No
	*DCTN1*	74590116	NM_004082.4	c.3529 + 5G > A^†^		-	-	0.59160	No
	*SERPINI1*	167512569	NM_001122752.1	Gca/Aca	Ala280Thr	tol	ben	1.125	No

*Sift*, Sorting Intolerant From Tolerant software; *PolyPhen*, Polymorphism Phenotyping software; *GnomAD*, Genome Aggregation Database; *HGMD*, Human Gene Mutation Database; *ben*, benign; *del*, deleterious; *dam*, damaging; *poss*, possibly; *prob.*, probably; *tol*, tolerated

*Combined annotation dependent depletion (CADD) score (https://cadd.gs.washington.edu/) was > 35

^†^NetGene2 (http://www.cbs.dtu.dk/services/NetGene2/) and BDGP (http://www.fruitfly.org/seq_tools/splice.html) splice site prediction tools predicted loss of splice site

Some variants have never been reported in any control (GnomAD) or disease (HGMD) databases, representing variants unique to our cases. CADD analysis of the *SORL1* Ser10STOP variant predicted pathogenicity, as well as NetGene2 and BDGP predictions of the *DCTN1* c.3529 + 5G > A splice variant.

## Discussion

Alzheimer’s disease is mainly distinguished in a typical presentation with hippocampal amnestic syndrome and atypical forms with different cognitive or behavioural deficits.

In this paper, we describe a series of 6 unrelated patients affected by dementing syndromes characterized by one or more “*atypical*” features including age at onset, clinical presentation and disease progression rate. Case 5 presented a complex syndrome indicative of bvFTD with parkinsonism and additional atypical features. The severity of clinical picture and the high levels of CSF tau might suggest the possibility of a prion disease. However, the long course, the MRI features, the neuroimaging findings (parieto-temporal atrophy and hypometabolism) and CSF Aβ_42_ reduction made presenile AD the most likely diagnosis. Case 6 was classified as possible CBS, a clinical syndrome with different underlying pathological substrates [11, 12]. In vivo AD pathophysiological biomarkers and ^18^FDG-PET hypometabolic pattern suggested an underlying AD pathology (CBS-AD), in agreement with the results of a recent combined ^18^FDG-PET/neuropathological study [13]. Notably, in all patients, the in vivo AD pathophysiological biomarkers supported the diagnosis of probable AD. Indeed, these biomarkers should always be looked for, together with the downstream degenerative topographical biomarkers (^18^FDG-PET, MRI), in atypical dementia cases.

The results of genetic analyses were, in our opinion, very interesting. The variant found in cases 1 and 2, *PSEN1* Glu318Gly, was first identified in patients with EOAD [14]. Studies performed to define its effects on amyloid-β metabolism gave conflicting results [15, 16], and association studies were inconclusive [16, 17]. The variant disclosed in case 3, *PSEN2* Arg71Trp, probably involved in protein stability and signalling pathways [18], has been found in patients with EOAD or LOAD, as well as in healthy subjects and Parkinson’s disease dementia [19, 20], and only in one large AD family it seemed to clearly segregate with the disease [21, 22]. It is possible that, by interacting with other factors, *PSEN1* Glu318Gly and *PSEN2* Arg71Trp increase disease risk and modulate clinical phenotype. *PSEN2* Ser236Ser, present in case 4, is a silent variant whose pathogenicity is not predictable.

Among the relevant findings of NGS analysis, *ABCA7* and *SORL1* are well-known AD risk genes [23, 24]. The ABCA7 transporter is involved in Aβ clearance and its mutations accelerate amyloidosis in a mouse model of AD [25]. A strong association was demonstrated between ABCA7 variations and amyloidosis in AD patients [26]. A reduced expression of SORL1, promoter of the APP non-amyloidogenic pathway [27], has been demonstrated in human AD brains, and its genetic variants increase risk of both LOAD and EOAD [28]. In patient 1, we identified the *ABCA7* Asp679Tyr and the *SORL1* Thr833Ile variants. They had never been reported before but are predicted to be deleterious by in silico analyses, therefore possibly exerting a synergistic effect with the *PSEN1* Glu318Gly variant in amyloidogenic process.

Patient 2, affected by LOAD with parkinsonism, harboured the Tyr239Cys variant in *FUS*, a gene implicated in ALS and FTD cases [29]. This variant is present in GnomAD with a very low frequency and is predicted to be deleterious by some in silico analyses.

In patient 5, we found the Iso196Val variant in *DCTN1* gene. Several *DCTN1* mutations have been described in association with ALS, degenerative parkinsonisms and Perry syndrome [30, 31]. Interestingly, our patient displayed some features of Perry syndrome at disease onset, such as personality change, and eating and sleep disturbances, while parkinsonism occurred thereafter. However, in vivo biomarkers more likely predicted amyloid-β rather than TDP-43 pathology, which is Perry syndrome’s substrate. Despite some evidence of pathogenicity from in vitro studies [32], *DCTN1* Iso196Val variant has been reported both in patients and in several healthy controls, making it a possible risk factor rather than a causative mutation. This patient also presented the Ser10STOP variant in *SORL1*, which is a truncating variant absent in ExAc (Exome Aggregation Consortium, http://exac.broadinstitute.org/) and GnomAD databases, with a CADD score of 35: these types of variant are considered as definitely pathogenic and associated with a significant 12-fold increased AD risk, which is comparable with the *APOE*-ε4 homozygosity effect [33]. Rare pathogenic *SORL1* mutations segregate with disease in LOAD families, and their pathological mechanism is likely to be haploinsufficiency [34].

In case 6, we found variants in other dementia-related genes. The novel Ala273Thr variant, predicted as damaging by in silico analysis, was identified in *CSF1R*. *CSF1R* mutations are causative of adult-onset leukoencephalopathy with axonal spheroids and pigmented glia [35], and have recently been reported in pathologically confirmed AD subjects [36]. Noteworthy, one of these cases exhibited a clinical picture very similar to that of our case. We can therefore hypothesize that rare variants of *CSF1R* may influence the susceptibility to AD, as already shown for other adult-onset leukodystrophy causative genes, such as *TREM2* and *NOTCH3* [37, 38]. Mutations in *SERPINI1* are responsible for familial encephalopathy with neuroserpin inclusion bodies [39]. Though rapidly progressive dementia and myoclonus belong to the clinical spectrum of *SERPINI1* mutations [40], the Ala280Thr variant found in patient 6 is predicted as tolerated by in silico analyses. Finally, the splicing mutation c.3529 + 5G > A identified in *DCTN1* gene is predicted as potentially capable of altering the splicing site by in silico analyses; therefore, a possible pathogenic effect cannot be excluded.

In conclusion, two relevant aspects emerge from the observations made on this case series. First, some of the patients here presented are paradigmatic of the difficulties in reaching a confident in vivo diagnosis due to the “*atypical*” clinical aspects, despite the application of very extensive diagnostic protocols. Therefore, post-mortem neuropathological examination remains the gold standard to definitely elucidate the nature of the neurodegenerative process in the single patient with atypical dementia.

Second, in this series of cases, it is also possible to highlight the very interesting aspects emerging from a wider than standard genetic analysis. We found the coexistence of more than one rare non-causative genetic variant in 4 out of 6 patients, suggesting an additive contribution of them to develop dementia, whereas each single variant may not be sufficient. This raises a crucial question: what is the role of these non-causative mutations that are increasingly found in different neurological disorders, particularly in dementias? One hypothesis is that they could act as risk or modifier factors to the disease. Further studies adding evidence from NGS data to the current knowledge will be necessary to support this hypothesis and to define the individual risk associated to each variant.

## Data Availability

There are no figures, videos or other data which could allow the identification of the subjects.
